# Mechanical vs. Operative Dentistry

**Published:** 1875-08

**Authors:** P. T. Smith


### MECHANICAL *VS.* OPERATIVE DENTISTRY.

BY P. T. SMITH.

*Read before the Iowa State Dental Society.*

Separating mechanical from operative, or medical dentistry should claim our earnest attention, as in this change we are promised a new impetus in professional advancement.

It is intended in this to only mention in brief some of the most apparent reasons why such change should be made, thus opening the subject for discussion.

To the most casual observer and thinker, the divergent characteristics of mechanical and medical dentistry are so glaring that but little tenable argument can be offered for their continued association, and a conformity to the proofs for their separation, by each, only awaits the sanction of the whole, when we shall enjoy the promises of many of our happy anticipations, in the relief from contending elements in different natures.

The more we lineate in our educational efforts the greater perfection we may attain, as the field of the dental physician proper, is ample for the most comprehensive mental powers. The plea for concentrated effort in that future line is the more potent, since its association with mechanical dentistry not only retards progress by theft of time, but stultifies by contamination. Evidence for this change is prominent in the fact that obtains in all, but perhaps more especially with the American people, to turn every moment of time and material thing possible to a financial substantiality, thus rendering mechanical, as an alliance to operative dentistry, particularly dangerous to the latter, on account of the comparatively easy manner the manipulative knowledge of the former may be gained—establishing and multiplying its votaries on every hand—from that class of young men being literary, and perhaps financial objections, on account of the facts secured to them by these particular reasons.

The professional literary standard of the members of the dental profession is regarded to-day, at not a very high elevation, and admitted by members of the kindred professions with a spirit of interdiction, on account of our trammeled position by the overwhelming influence of the mechanical association and its inevitable tendencies.

A young man without much literary preparation may enter —as many do—the laboratory of some "dental surgeon" and acquire in six months time, a sufficient knowledge of the the business to pass himself acceptably with the people, fully meeting their expectations or satisfying their limited ideas of what a dentist should be.

Able to extract teeth, make new sets on some disagreeable substance, and occasionally stuff one with amalgam, the mechanical part of the business being the great beacon light of dental perfection held up to view—to allure the victims on to destruction and death, making the way clear to the wiry practitioner to set up porcelain tomb stones to mark the place of the sacred and lamented organs so ruthlessly sacrificed and torn from their attachments in physical economy. All on account of ignorance and love of money.

Educate the dental student to treat diseases and save the human teeth, encourage him to do what he can to prevent disease, and by his efforts and influence, the people will fast become acquainted with their first duties, and no longer be subject to that charlatanic influence that to-day sees first of all, and encourages their credulous victims to compliance in the ruthless slaughter of their teeth in the furtherance of their insatiate money greed.

Give us the change. Let us become, as the necessities require, purely professional, and we have nothing in our way to that coveted recognition that we now hardly dare claim to merit. Also we relieve ourselves in the speediest and most effectual way of the annoyances the Legislature has so often refused to interfere in.

It will give us renewed strength and ability in the channels of most importance to our patients—that is teaching to them hygenic laws and their obedience to them, as a means of better health and teeth; to us, better success in our efforts, better prices and better feelings among men of the profession.

I would cast no reflection upon the usefulness and honor of mechanical efforts and the unfortunate necessities of the mechanical dentist but would encourage a position for such as may enjoy what that department in its properly circumscribed arena may offer. Such a place as will afford its votaries no opportunity of enlarging their work by the destruction of sacred organs that in the hands of the learned and skilled could be saved and continued in their office of vital perpetuity.

